# A Retrospective Review of the Impact of Racial Disparities and Outcomes on Urinary Anastomotic Leak in Radical Prostatectomy in a National Surgical Registry

**DOI:** 10.7759/cureus.80836

**Published:** 2025-03-19

**Authors:** Benjamin A Fink, Young Son, Kimberly C Toumazos, Virgil K DeMario, Frederick Okoye, Ryan Moriarty, Thomas J Mueller

**Affiliations:** 1 Urology, Jefferson Stratford Hospital, Stratford, USA; 2 Department of Clinically Applied Science Education, University of the Incarnate Word School of Osteopathic Medicine, San Antonio, USA; 3 Clinical Research, Philadelphia College of Osteopathic Medicine, Philadelphia, USA; 4 Urology, New Jersey Urology, LLC, Voorhees, USA

**Keywords:** prostate cancer (pca), prostatectomy complications, racial disparities, radical prostatectomy, urinary anastomotic leak (ual)

## Abstract

Background

Racial disparities exist in prostate cancer incidence and mortality. Radical prostatectomy, a common treatment for prostate cancer, has been associated with increased complications in African American men compared to other racial groups. An anastomotic urinary leak is associated with prolonged hospitalization rates, increased rates of ileus, and a longer time to regain continence. This analysis aims to evaluate differences in urinary anastomotic leak (UAL) rates between racial groups and potential predictive factors for this disparity.

Methodology

The American College of Surgeons National Surgical Quality Improvement Program database (2019-2020) was utilized to compare rates of UAL in African American versus White patients. Preoperative variables examined included patient demographics, comorbid conditions, and surgical preparation. Other variables assessed included medical complications (such as pulmonary embolism), postoperative diagnosis, and surgical complications.

Results

A total of 11,010 radical prostatectomy patients were analyzed after applying selection criteria. African American men were more likely to be younger, current smokers, and on dialysis. African American men were also more likely to have had prior pelvic radiotherapy, diabetes mellitus, dyspnea, and hypertension controlled with medications. The odds of having a UAL in African American men after radical prostatectomy were 51% higher than in Whites (odds ratio = 1.51, 95% confidence interval = 1.02-2.17, p = 0.032). Prior pelvic surgery, chemotherapy within 90 days, mean operative time, and readmission rates were all associated with UAL in African American men postoperatively.

Conclusions

There is a significant disparity in the rate of UAL in African American versus White men and multiple factors may influence this difference.

## Introduction

Prostate cancer is one of the most common malignancies and causes of cancer death among men [1]. In the United States, racial disparities exist in prostate cancer incidence and mortality rates. There is a significantly increased rate of prostate cancer among African American men (183.4 cases per 100,000) compared to White (110.0 cases per 100,000) or Asian (59.6 cases per 100,000) men [1]. African American men are more likely to be diagnosed with prostate cancer at an earlier age and with more advanced or metastatic disease when compared to other racial groups [2,3]. This stark racial disparity is best highlighted by the fact that prostate cancer mortality among African American patients is significantly elevated when compared to White patients [4]. The factors that contribute to this gap are of significance in comparison to the differences in the incidence of prostate cancer and demand exploration into potential influences that may lie within current patterns in screening, treatment modalities, and complication rates.

One of the gold-standard treatments for patients with intermediate- or high-risk clinically localized prostate cancer is radical prostatectomy. However, this procedure has been associated with increased rates of unadjusted Clavien III and V complications compared to other racial groups, including venous thromboembolism in African American men [5,6]. Urinary leak at the urethrovesical anastomosis most commonly diagnosed via cystogram has been cited as a complication of radical prostatectomy, with incidence rates as high as 15% [7-9]. Urinary anastomotic leak (UAL) is associated with increased rates of ileus, prolonged hospitalization rates, and longer time to achieve continence [10,11]. Furthermore, other short-term comorbidities such as urinary retention and sepsis have been reported [12]. Various risk factors have been identified, including increased body mass index (BMI) and prior abdominal surgery [11].

Although the current literature has not addressed racial disparities in UAL, studies have outlined relationships between demographics, including race, in other types of anastomotic leaks. For instance, one study identified race as a significant factor in gastrointestinal anastomotic leak in that African American patients were more likely to have a leak when compared to White patients [13].

There are likely several patient and surgical factors that contribute to the development of UAL following radical prostatectomy; however, the extent to which race is a risk factor for this complication has not been previously well evaluated. In our study, we used the American College of Surgeons National Surgical Quality Improvement Program (ACS NSQIP) database to compare the rates of post-radical prostatectomy UAL between African American and White patients. Preoperative demographics with comorbidities and postoperative outcomes were analyzed.

The objective of this study is to determine the impact of the racial group on rates of UAL in patients who underwent radical prostatectomy for malignant neoplasm of the prostate. The goal is to also identify if pathological staging and comorbid conditions contribute to UAL in African American patients to provide better counseling for patients. Secondary aims include predicting independent risk factors associated with UAL in African American patients.

## Materials and methods

Data source

The ACS NSQIP is a Health Insurance Portability and Accountability Act-compliant data file containing patient cases from 706 participating hospital institutions. This data set includes variables on 902,968 cases in 2020 and 273 variables on 1,076,441 cases in 2019. ACS NSQIP includes principal operative procedure cases as determined by Current Procedural Terminology codes 55801, 55810, 55812, 55815, 55821, 55831, 55840, 55842, 55845, 55866. The goal of the program is to determine the quality of care after surgical procedures. The ACS NSQIP is de-identified and therefore this retrospective study was deemed exempt from the institutional review board.

Study population and definitions

**Figure 1:**
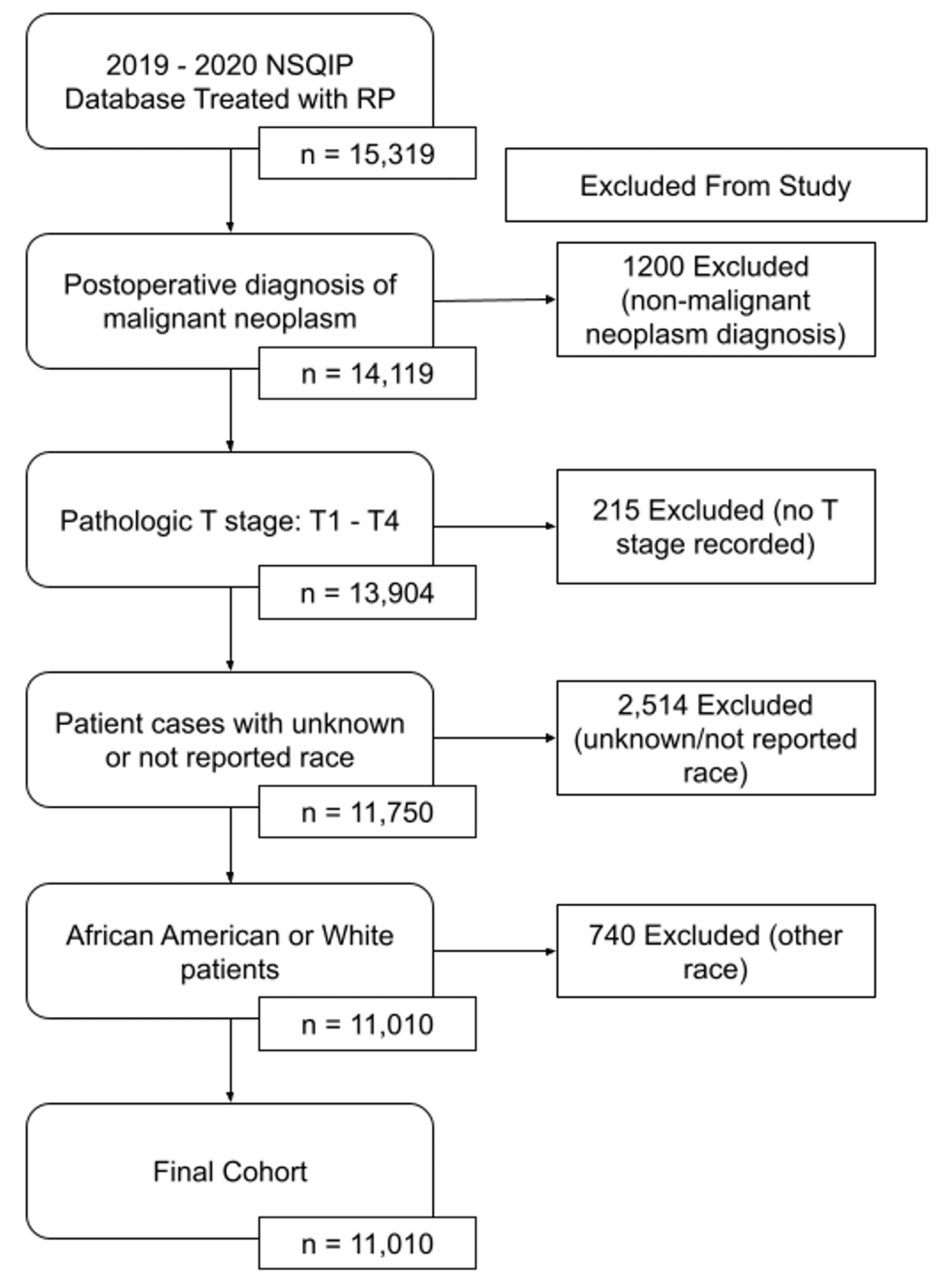
CONSORT flow diagram of the study cohort. NSQIP: National Surgical Quality Improvement Program; RP: radical prostatectomy

We analyzed the 2019 and 2020 NSQIP database for patients who underwent radical prostatectomy for prostate cancer. A total of 15,319 patients who underwent radical prostatectomy were initially screened in the NSQIP database. Inclusion criteria included a postoperative diagnosis of malignant neoplasm of the prostate, personal history of malignant neoplasm of the prostate, carcinoma in situ of the prostate, neoplasm of uncertain behavior of the prostate, and atypical small acinar proliferation of the prostate. A CONSORT flow diagram displays our study sample (Figure 1). Patients without data available for a postoperative diagnosis were excluded. Patients were then divided into different categories secondary to their race. White race was defined by NSQIP as: “A person having origins in any of the original peoples of Europe, the Middle East, or North Africa” and African American was defined as: “A person having origins in any of the black racial groups of Africa. Terms such as ‘Haitian’ can be used in addition to ‘Black’ or ‘African American.’” Patient cases with unknown or not reported race were excluded. Races including American Indian or Alaska Native, Asian, Native Hawaiian or Other Pacific Islander, Native Hawaiian or Pacific Islander, and Some other Race were also excluded. UAL was defined as persistent drainage (output of any significant volume of fluid on or after postoperative day three) with one of the following criteria: drain continued longer than seven days, spontaneous wound drainage, or additional invasive interventions required.

Perioperative, intraoperative, and postoperative variables were analyzed between the White and the African American groups, in addition to patients being subdivided by surgical approach. In terms of variables, surgical site infection was defined as either a superficial, deep, or organ-space infection. All staging was considered pathologic T, N, or M stages. Transfer status as defined by “not transferred - admitted by home,” was used to represent patients who were admitted to the hospital directly from home. Functional health status before surgery was set as comparable to a score from Eastern Cooperative Oncology Group Performance Status of 0-2. In terms of prior pelvic surgery, the specific surgeries included in this variable and the amount of time before prostatectomy could not be defined.

For the open wound variable, the type of wound and/or associated infection could not be identified in addition to the preoperative and postoperative hemoglobin, nor the type of bleeding included in the intraoperative bleeding variable. The decision to operate in lieu of radiation therapy was not available for analysis within this data set. Regarding the mean operative time, NSQIP defined operative time as the total time a patient spends in the operating room, from the start of surgery to the end. The NSQIP defines superficial incisional infections as those associated with purulent drainage, positive culture, pain, tenderness, swelling, erythema, or warmth. Deep incisional infections are defined as those including purulent drainage, deep incisions that dehisce or are opened, abscesses, or infections found on direct examination, reoperation, histology, or imaging. Organ-space infections are defined as expanding purulent drainage from a drain, positive culture from an aseptically placed drain, abscesses, or infections found on direct examination, reoperation, histology, or imaging.

Furthermore, the African American cohort was subdivided into those who had a UAL versus those who did not. The two groups were then analyzed to predict the independent variables associated with UAL in African American men.

Statistical analysis

The statistical analysis was performed between the White and African American cohorts and African American UAL group and African American without UAL group. For categorical variables, a chi-squared test was performed. One-sample t-test was used for continuous variables. Additionally, for selected statistically significant data of interest, odds ratios (ORs) were reported where appropriate. Univariate and multivariate logistic regression were performed. The random forest algorithm was performed to classify significant predictors by their predictive power to generate importance measures. Variables demonstrating statistical significance in multivariate analysis (p < 0.05) and those deemed clinically relevant were considered for inclusion in the multivariable model. These importance measures in conjunction with clinical judgment were used to determine the independent variables for the multivariate logistic model as well as potential confounders. Statistical analysis was performed using R version 4.0.2 (R Foundation for Statistical Computing, Vienna, Austria). Statistical significance was accepted at a p-value <0.05.

## Results

A total of 15,319 patients were reviewed and a final cohort of 11,010 patients were included in the retrospective study (Table 1). Among the study population, 1,737 were African American and 9,273 were White. The overall UAL rate for the total patient cohort was 1.56%, with 2.30% in African Americans and 1.42% in Whites (p = 0.00914) (Table 2). The odds of UAL in African American men were 51% higher than in Whites (OR = 1.51, 95% confidence interval (CI) = 1.02-2.17, p = 0.032) (Table 3).

**Table 1:** Preoperative characteristics and comorbidities of African American versus White patients. SD: standard deviation; COPD: chronic obstructive pulmonary disease; HTN: hypertension; PRBC: packed red blood cells; ASA: American Society of Anesthesiologists; BUN: blood urea nitrogen; PTT: partial thromboplastin time; INR: international normalized ratio

	Total cohort (mean ± SD)	African American (mean ± SD)	White (mean ± SD)	P-value
	n = 11,010	n = 1,737	n = 9,273	
Patient characteristics				
Mean patient age (SD)	63.2 ± 6.79	60.9 ± 7.11	63.7 ± 6.64	0.001
Height (in) (SD)	68.702 ± 11.1	68.6 ± 11.1	68.7 ± 11.1	0.771
Weight (lbs) (SD)	201 ± 37.9	204 ± 42.1	200 ± 37.1	0.001
Past medical history/comorbidities				
Current smoker (within one year) (%)	1,144 (10.4%)	318 (18.3%)	826 (8.91%)	0.001
Transfer status (not transferred - admitted from home) (%)	10,984 (99.8%)	1,723 (99.2%)	9,261 (99.9%)	0.001
Functional health status before surgery (independent) (%)	10,993 (99.8%)	1,730 (99.6%)	9,263 (99.9%)	0.00375
Prior pelvic surgery (%)	1,880 (17.1%)	227 (13.1%)	1,653 (17.8%)	0.001
Prior pelvic radiation (%)	78 (0.708%)	22 (1.27%)	56 (0.604%)	0.00416
Chemotherapy within 90 days (%)	99 (0.899%)	13 (0.748%)	86 (0.927%)	0.557
Preoperative oral antibiotic prep (%)	393 (3.57%)	53 (3.05%)	340 (3.67%)	0.001
Perioperative antibiotic use (<72 hours) (%)	763 (6.93%)	132 (7.6%)	631 (6.8%)	0.04
Mortality (%)	4 (0.0363%)	0 (0%)	4 (0.0363%)	1.00
Comorbid conditions				
Diabetes with insulin dependency (%)	10,656 (96.8%)	1,627 (93.7%)	9,029 (97.4%)	<0.001
Diabetes with oral agents (%)	9,870 (89.6%)	1,449 (83.4%)	8,421 (90.8%)	<0.001
Congestive heart failure (%)	15 (0.136%)	4 (0.23%)	11 (0.119%)	0.422
Severe COPD (%)	195 (1.77%)	40 (2.3%)	155 (1.67%)	0.0834
HTN requiring meds (%)	5,834 (53%)	1,150 (66.2%)	4,864 (52.5%)	0.132
Steroid use for chronic conditions (%)	219 (1.99%)	26 (1.5%)	193 (2.08%)	0.001
Dyspnea (%)	248 (2.25%)	53 (3.05%)	195 (2.1%)	0.029
Currently on dialysis (preoperative) (%)	35 (0.318%)	16 (0.921%)	19 (0.205%)	0.001
Bleeding disorders (%)	110 (1%)	16 (0.921%)	94 (1.01%)	0.822
>10% body weight loss in the last six months (%)	13 (0.118%)	3 (0.173%)	10 (0.108%)	0.732
Transfusion ≥1 units of PRBC 72 hours before surgery (%)	3 (0.0272%)	3 (0.173%)	0 (0%)	0.00133
Malignancy stage (pathologic)				
T1 (T1a, T1b, T1c) (%)	36 (0.327%)	6 (0.345%)	30 (0.324%)	1.00
T2 (T2a, T2b, T2c) (%)	6,313 (57.3%)	1,058 (60.9%)	5,255 (56.7%)	0.00114
T3 (T3a, T3b, T3c) (%)	4,644 (42.2%)	670 (38.6%)	3,974 (42.9%)	0.001
T4 (%)	17 (0.154%)	3 (0.173%)	14 (0.151%)	1.00
ASA classification				
Class 1 (%)	225 (2.04%)	25 (1.44%)	200 (2.16%)	0.0647
Class 2 (%)	5,907 (53.7%)	818 (47.1%)	5,089 (54.9%)	0.001
Class 3 (%)	4774 (43.4%)	874 (50.3%)	3,900 (42.1%)	0.001
Class 4 (%)	100 (0.908%)	20 (1.15%)	80 (0.863%)	0.305
Serum values				
Elevated sodium (≥146 mEq/L) (%)	51 (0.463%)	0 (0%)	51 (0.55%)	0.737
Elevated BUN (≥21 mg/dL) (%)	1,906 (17.3%)	33 (1.9%)	1,873 (20.2%)	0.580
Elevated PTT (≥36 seconds) (%)	175 (1.59%)	4 (0.23%)	171 (1.84%)	0.638
Elevated INR (≥1.16) (%)	201 (1.8%)	6 (0.345%)	195 (2.1%)	0.176

**Table 2:** Perioperative and postoperative outcomes and complications in African American versus White patients. SD: standard deviation; CVA: cerebrovascular accident; CPR: cardiopulmonary resuscitation; DVT: deep vein thrombosis; NPO: nothing by mouth; NGT: nasogastric tube; SSI: surgical site infection

	Total cohort (mean ± SD)	African American (mean ± SD)	White (mean ± SD)	P-value
	n = 11,010	n = 1,737	n = 9,273	
Operative approach				
Mean operative time (SD)	209.67 ± 69.561	225 ± 73.678	207 ± 68.4	0.001
Planned laparoscopic/Robotic (%)	8,141 (73.9%)	1,323 (76.1%)	6,818 (73.5%)	0.0231
Planned open (%)	825 (7.49%)	111 (6.39%)	714 (7.7%)	0.0639
Unplanned conversion to open (%)	15 (0.136%)	1 (0.0576%)	14 (0.151%)	0.539
Mean days from operation to discharge (SD)	1.3 ± 1.57	1.52 ± 1.82	1.26 ± 1.51	0.001
Mean number of removed lymph nodes evaluated (SD)	4.61 ± 18.7	3.47 ± 20.9	4.82 ± 18.2	0.00576
Readmission (%)	448 (4.07%)	80 (4.61%)	368 (3.97%)	0.243
Postoperative complications				
Urinary anastomotic leak (%)	172 (1.56%)	40 (2.3%)	132 (1.42%)	0.00914
Ureteral obstruction (%)	53 (0.481%)	12 (0.691%)	41 (0.442%)	0.114
Blood transfusion (%)	286 (2.6%)	38 (2.19%)	248 (2.67%)	0.236
Sepsis (%)	63 (0.572%)	12 (0.691%)	51 (0.55%)	0.276
Septic shock (%)	9 (0.0817%)	3 (0.173%)	6 (0.0647%)	0.589
Urinary tract infection (%)	238 (2.16%)	53 (3.05%)	185 (2%)	0.323
Acute renal failure (%)	3 (0.0272%)	1 (0.0576%)	2 (0.0216%)	0.00719
CVA/Stroke with a neurological deficit (%)	3 (0.0272%)	1 (0.0576%)	2 (0.0216%)	0.966
Cardiac arrest requiring CPR (%)	9 (0.0817%)	4 (0.23%)	5 (0.0539%)	0.966
Myocardial infarction (%)	16 (0.145%)	1 (0.0576%)	15 (0.162%)	0.0571
Pulmonary embolism (%)	68 (0.618%)	7 (0.403%)	61 (0.658%)	0.482
DVT/Thrombophlebitis (%)	70 (0.636%)	10 (0.576%)	60 (0.647%)	0.281
Discharge to home (%)	10,931 (99.3%)	1,710 (98.4%)	9,221 (99.4%)	0.858
Rectal injury (%)	35 (0.318%)	5 (0.288%)	30 (0.324%)	0.001
Unplanned intubation (%)	15 (0.136%)	6 (0.345%)	9 (0.0971%)	0.992
Wound disruption (%)	13 (0.118%)	1 (0.0576%)	12 (0.129%)	0.675
*Clostridium difficile* colitis (%)	13 (0.118%)	1 (0.0576%)	12 (0.129%)	0.675
Prolonged postoperative NPO or NGT use (%)	128 (1.16%)	43 (2.48%)	85 (0.917%)	0.001
Lymphocele, lymphatic leak, or other fluid collection (%)	199 (1.81%)	33 (1.9%)	166 (1.79%)	0.828
Unplanned reoperation related to the principal procedure (%)	124 (1.13%)	26 (1.5%)	98 (1.06%)	0.141
SSI				
Superficial SSI (%)	86 (0.781%)	13 (0.748%)	73 (0.787%)	0.984
Deep incisional SSI (%)	3 (0.0272%)	0 (0%)	3 (0.0272%)	1.00
Organ-space SSI (%)	116 (1.05%)	23 (1.32%)	93 (1%)	0.282

**Table 3:** Multivariate logistic regression analysis examining factors associated with the African American cohort. OR: odds ratio; CI: confidence interval; ASA: American Society of Anesthesiologists

	Univariate analysis			Multivariate analysis		
Variable	OR	95% CI	P-value	OR	95% CI	P-value
Patient age	0.94	0.94–0.95	0.001	0.93	0.93–0.94	<0.001
Diabetes with insulin dependency (%)	2.5	1.98–3.14	<0.001	2.56	2.00–3.26	<0.001
Diabetes with oral agents (%)	1.96	1.70–2.27	<0.001	2.11	1.81–2.46	<0.001
Steroid use for chronic conditions (%)	0.71	0.46–1.06	0.111	0.65	0.42–0.97	0.046
Currently on dialysis (preoperative) (%)	4.53	2.30–8.82	<0.001	3.37	1.63–6.88	0.001
Mean operative time	1	1.00–1.00	<0.001	1	1.00–1.00	<0.001
Mean length of stay	1.06	1.03–1.10	0.001	1.03	1.00–1.06	0.130
Urinary anastomotic leak	1.63	1.13–2.31	0.007	1.51	1.02–2.17	0.032
ASA classification						
Class 1 (%)		Referent			Referent	
Class 2 (%)	0.73	0.66–0.81	<0.001	1.39	0.92–2.19	0.130
Class 3 (%)	1.4	1.26–1.55	<0.001	1.83	1.21–2.88	0.006
Class 4 (%)	1.34	0.80–2.14	0.246	1.86	0.94–3.65	0.073

The African American population was younger (60.9 years vs. 63.7, p = 0.001), more likely to be a current smoker (18.3% vs. 8.91%, p = 0.001), had higher prior pelvic radiation (1.27% vs. 0.604%, p = 0.00416), and currently on dialysis (0.921% vs. 0.205%, p = 0.001). Although neoadjuvant chemotherapy is not a regimented treatment for prostate cancer, we observed that 0.748% of African American patients received chemotherapy within 90 days of prostatectomy, presumably for another malignancy or autoimmune process. Perioperatively, African American prostatectomies were longer (225 minutes vs. 207 minutes). Postoperatively, African American men who had undergone radical prostatectomy averaged a longer hospital stay (1.52 days vs. 1.26 days, p = 0.001), acute renal failure (0.0576% vs. 0.0216%, p = 0.00719), and prolonged postoperative nothing by mouth (NPO) or nasogastric tube (NGT) use (2.48% vs. 0.917%, p = 0.001).

Univariate, multivariate, and bivariate analyses showed that prior pelvic surgery (OR = 5.25, 95% CI = 0.93-20.78, p = 0.033), chemotherapy within 90 days (OR = 12.69, 95% CI = 2.50-54.35, p = 0.001), and mean operative time (OR = 1.01, 95% CI = 1.00-1.01, p = 0.004) were all independent factors that lead to UAL in the African American cohort. Additionally, readmission rates (OR = 5.69, 95% CI = 1.95-14.81, p = 0.001) and ureteral obstruction (OR = 32.26, 95% CI = 7.26-132.86, p < 0.001) were associated with UAL postoperatively (Tables 4, 5). Mean days from operation to discharge; prolonged postoperative NPO or NGT use; lymphocele, lymphatic leak, or other fluid collection; and organ-space surgical site infection were not significantly associated with UALs in the African American cohort when controlling in the multivariate analysis; however, it was significant in the univariate analysis.

**Table 4:** Postoperative outcomes and complications in African Americans with urinary anastomotic leak versus no leak. SD: standard deviation; CVA: cerebrovascular accident; CPR: cardiopulmonary resuscitation; DVT: deep vein thrombosis; NPO: nothing by mouth; NGT: nasogastric tube; SSI: surgical site infection

	Total cohort (mean ± SD)	Urinary anastomotic leak (mean ± SD)	No urinary anastomotic leak (mean ± SD)	P-value
	n = 1,737	n = 40	n = 1,697	
Operative approach				
Planned laparoscopic/Robotic (%)	1,323 (76.2%)	29 (72.5%)	1,294 (76.3%)	0.717
Planned open (%)	111 (6.39%)	2 (5%)	109 (6.42%)	0.971
Unplanned conversion to open (%)	1 (0.0576%)	0 (0%)	1 (0.0589%)	1.00
Outcome variables				
Mean operative time (SD)	224.99 ± 73.7	262.13 ± 88.1	224.11 ± 73.1	0.00124
Mean length of stay (SD)	1.42 ± 3.87	3.15 ± 3.87	1.37 ± 3.86	0.00414
Mean days from operation to discharge (SD)	1.52 ± 1.82	3.15 ± 3.87	1.48 ± 1.73	<0.001
Mean number of nodes evaluated (SD)	3.47 ± 20.9	9.38 ± 8.08	3.33 ± 21.1	0.0711
Readmission (%)	80 (4.61%)	13 (32.5%)	67 (3.95%)	<0.001
Postoperative complications				
Urinary anastomotic leak (%)	40 (2.3%)	40 (100%)	0 (0%)	<0.001
Ureteral obstruction (%)	12 (0.691%)	6 (15%)	6 (0.354%)	<0.001
Blood transfusion (%)	38 (2.19%)	4 (10%)	34 (2%)	0.00410
Sepsis (%)	12 (0.691%)	3 (7.5%)	9 (0.53%)	<0.001
Septic shock (%)	3 (0.173%)	0 (0%)	3 (0.177%)	1.00
Urinary tract infection (%)	53 (3.05%)	3 (7.5%)	50 (2.94%)	0.234
Acute renal failure (%)	1 (0.0576%)	0 (0%)	1 (0.0589%)	1.00
CVA/Stroke with neurological deficit (%)	1 (0.0576%)	0 (0%)	1 (0.0589%)	1.00
Cardiac arrest requiring CPR (%)	4 (0.23%)	0 (0%)	4 (0.236%)	1.00
Myocardial infarction (%)	1 (0.0576%)	0 (0%)	1 (0.0589%)	1.00
Pulmonary embolism (%)	7 (0.403%)	1 (2.5%)	1,691 (99.6%)	0.392
DVT/Thrombophlebitis (%)	10 (0.576%)	1 (2.5%)	9 (0.53%)	0.568
Discharge to home (%)	1,710 (98.4%)	40 (100%)	1,670 (98.4%)	0.875
Rectal injury (%)	5 (0.288%)	0 (0%)	5 (0.295%)	1.00
Unplanned intubation (%)	15 (0.864%)	6 (15%)	9 (0.53%)	0.0100
Wound disruption (%)	1 (0.0576%)	0 (0%)	1 (0.0589%)	1.00
*Clostridium difficile* colitis (%)	1 (0.0576%)	0 (0%)	1 (0.0589%)	1.00
Prolonged postoperative NPO or NGT use (%)	43 (2.48%)	9 (22.5%)	34 (2%)	<0.001
Lymphocele, lymphatic leak, or other fluid collection (%)	33 (1.9%)	5 (12.5%)	28 (1.65%)	<0.001
Unplanned reoperation related to principal procedure (%)	26 (1.5%)	8 (20%)	18 (1.06%)	<0.001
SSI				
Superficial SSI (%)	13 (0.748%)	0 (0%)	13 (0.766%)	1.00
Deep incisional SSI (%)	0 (0%)	0 (0%)	0 (0%)	N/A
Organ-space SSI (%)	23 (1.32%)	8 (20%)	15 (0.884%)	<0.001

**Table 5:** Multivariate logistic regression analysis examining predictors of urinary anastomotic leak. Variables demonstrating statistical significance in bivariate analysis (p < 0.05) and those deemed clinically relevant were considered for inclusion in the multivariable model. OR: odds ratio; CI: confidence interval; NPO: nothing by mouth; NGT: nasogastric tube; SSI: surgical site infection

	Univariate analysis			Multivariate analysis		
Variable	OR	95% CI	P-value	OR	95% CI	P-value
Prior pelvic surgery	7.16	1.63–22.19	0.002	5.25	0.93–20.78	0.033
Chemotherapy within 90 days	20.84	5.45–67.28	<0.001	12.69	2.50–54.35	0.001
Operative time	1.01	1.00–1.01	0.001	1.01	1.00–1.01	0.004
Days from operation to discharge	1.18	1.09–1.26	<0.001	1.11	0.98–1.24	0.077
Readmission	11.71	5.63–23.33	0.001	5.69	1.95–14.81	0.001
Ureteral obstruction	49.74	14.87–166.76	<0.001	32.26	7.26–132.86	<0.001
Prolonged postoperative NPO or NGT use	14.2	5.98–31.15	<0.001	0.88	0.21–3.42	0.864
Lymphocele/Lymphatic leak/Other fluid collection	8.52	2.77–21.69	<0.001	2.57	0.69–8.17	0.130
Organ-space SSI	28.03	10.64–69.52	<0.001	2.82	0.71–10.63	0.130

## Discussion

Significant racial disparities exist between African American and White patients with prostate cancer. In this study, we present evidence of an increased risk for UAL following radical prostatectomy in African American patients. Specifically, we found that the odds of an anastomotic leak were approximately 51% greater in African American than White patients. Our findings constitute an addition to the existing literature surrounding racial disparities in prostate cancer diagnoses and disease-state outcomes. The identification of racial disparities in healthcare, as well as actionable factors underlying them, is the first step in devising strategies to close these gaps and move toward a more equitable healthcare system.

We found that the development of UAL was associated with chemotherapy within 90 days of radical prostatectomy. We suspect the cytotoxic effects on cellular proliferation and turnover of rapidly dividing cells such as those found in urothelium may contribute to impaired healing of anastomotic sites, particularly in chemotherapies where this is more pronounced such as cyclophosphamide, cisplatin, and doxorubicin. The cellular toxicity imparted by chemotherapy resulting in tissue necrosis and damage has been well-described and associated with prolonged wound healing time [14]. This variation pertains directly to urologic procedures, including a significantly increased observed overall survival disparity, and the aforementioned Clavien III and V complications, among African American men undergoing radical prostatectomy compared to Whites [5,15].

Other mechanisms that may contribute to the higher rates of UAL in African American patients include ureteral obstruction, postoperative bleeding, infections, antibiotic prophylaxis, presence of hematomas, and surgical technique. Urine extravasation has been cited as a common complication of ureteral obstruction, frequently from forniceal rupture [16]. This leak may be affected by the release of inflammatory cytokines and other chemical mediators to the surrounding tissues, resulting in impaired wound healing, anastomotic failure, and eventual leak. Elevated creatinine and uremic conditions may also precipitate in the setting of urinary leak, causing delayed healing and platelet dysfunction [17]. As we display in the preoperative variables, African Americans were more likely to be on preoperative dialysis. Though we suggest obstruction as a possible mechanism for UAL, it is possible that ureteral obstruction may have also occurred secondary to an already-developed UAL, with a potentially developing urinoma causing extrinsic obstruction of the ureters. Dependent fluid collection collecting extravesicularly may also impart a mass effect on portions of the bladder and bladder neck, potentially causing similar inflammatory pathology, as well as compression of the blood supply to the healing urethral anastomosis, resulting in reduced healing.

Previous pelvic surgery was also associated with increased rates of UAL. Previous surgeries can induce an inflammatory response, resulting in impaired healing and leaks. Additionally, previous pelvic surgery could result in abdominal adhesions and alteration of the patient’s pelvic anatomy, making for a more technically challenging surgical landscape and decreasing viable tissue available to be used for, or support, the annealing of tissue and healing of the anastomosis. Although vague, the listed pelvic anatomy circumferentially envelopes the prostate and associated structures, and it would not be unreasonable to suggest that most laparoscopic or open pelvic surgery would have some effect on the regional tissues, ligaments, and visceral boundaries. Studies have shown that African American patients have a significantly greater sacral slope and pelvic incidence-lumbar lordosis when compared to White patients [18]. Although the robotic technique could mitigate such anatomic landscape versus the open and laparoscopic approaches, this study did not examine such differences.

Noteworthy secondary findings regarding demographic and preoperative characteristics included African American patients undergoing prostatectomy at a younger age and being more likely to have various comorbid conditions than White patients. Further highlighting the differences in baseline health status, African American patients were more likely than their White counterparts to have an American Society of Anesthesiologists (ASA) Class 3 and to have been transferred from another healthcare facility before surgery. Concerning postoperative considerations, mean operative time and mean length of stay were longer in the African American cohort. Complications after surgery, including acute renal failure, and prolonged postoperative NPO or NGT use were also elevated in the African American cohort. A thorough discussion of preoperative health and functional status is crucial to understanding why African American patients are disproportionately affected by UAL after radical prostatectomy. It is possible that the observed disparity is largely secondary to higher rates of tobacco use and dialysis requirement in the African American cohort. These disease states and their associated micro and macrovascular sequelae could all contribute to impaired healing of the vesicourethral anastomosis. Several studies have identified these aforementioned diseased states as risk factors for bowel anastomotic leak status post-cancer resection [19,20]. Prior investigations have identified risk factors for UAL, including ischemic heart disease, prior transurethral resection of the prostate, and increased blood loss [21]. Although the comorbidities we have discussed have not previously been established as risk factors for leaks, it is possible that having multiple systemic comorbidities, as reflected by an increased ASA classification and tobacco use, contribute to the development of this complication.

The observed differences in perioperative metrics and postoperative outcomes may also help explain the disparity in UAL between cohorts. For instance, there was a significantly longer operative time in the African American cohort. Previous studies have established that increased console time is a risk factor for the development of UAL [10]. As proposed by Kakutani et. al., it is reasonable to consider that increased operative time may be reflective of a more difficult surgery, including a more difficult and less stable anastomosis [10]. The statistically significant difference in readmission rates aligns with prior publications of urine leaks as a cause of rehospitalization [10].

Though one might consider the possibility that the difference in staging of disease between African American and White groups may have contributed to more technically challenging surgeries as the cause for the increased operative time, and thus, increased rates of complications inclusive of UAL, it appears that White patients had higher pathologic staging in our study. That African American patients had less advanced disease but still had increased adverse events and UAL among other characteristics and outcomes discussed is still further evidence of this racial disparity.

Limitations

Our study is limited due to its retrospective nature and the possibility of known biases common to this study type. In particular, the African American population of the ACS NSQIP database was not stratified to represent the diversity of the African diaspora. This includes the immigrant African population and non-first-generation African American patients. Because African ethnicity is not a monolith, further consideration in future studies may be considered for these potential differences. As BMI and rates of bladder neck contracture were also not reported as a finished data product in the ACS NSQIP, we were unable to draw conclusions relative to this variable. Our research offers a unique perspective on UAL and predictive factors that have not been covered in the literature. Although retrospective, the ACS NSQIP is a multi-institutional database with a large patient cohort, which offers significance to our findings. We also analyzed an extensive list of variables with univariate and multivariate analysis, effectively identifying the specific risk factors for UAL.

Future studies designed prospectively may better control for biases and potential confounding factors such as those discussed. Subsequent studies can also review additional postoperative outcomes other than UAL between racial groups. Now that UAL has been identified to occur more frequently in the African American population, extensive postoperative follow-up could be done to monitor the impact of UAL in this specific patient population. In terms of our study, a specific timeline of what occurred first, for instance, ureteral obstruction and UAL in conjunction with surgical approach, would improve the strength of our statistical analysis.

## Conclusions

A significant racial disparity exists in the incidence of UAL between African American and White patients. We have identified multiple variables that are increasingly associated with UAL in African American patients compared to White patients. In terms of our predicted objective that higher pathological staging and comorbid conditions contribute to UAL in African American patients, we did find that pathological stage was associated with a higher rate in African American patients, in addition to a potential association with comorbidities. By recognizing these factors, we aim to foster awareness in service of limiting these contributors to the wide racial disparity that we present.
